# Supplementary material for: Iterative improvement in the automatic modular design of robot swarms
Source: PeerJ Comput Sci. 2020 Dec 7;6:e322. doi: 10.7717/peerj-cs.322 (PMC7924708; doi:10.7717/peerj-cs.322)
Supplement: Supplemental Information 3 [file peerj-cs-06-322-s003.zip › argos3/doc/api/standalone/main.html]

ARGoS: Autonomous Robots GO Swarming


- Main Page
- Related Pages
- Namespaces
- Classes
- Files

# Autonomous Robots GO Swarming

### 3

**Author:**
:   Carlo Pinciroli <ilpincy@gmail.com>

**Version:**
:   3.0

**See also:**
:   http://iridia.ulb.ac.be/
:   http://www.swarmanoid.org/
:   http://ascens-ist.eu/

---

Generated on 10 Jul 2018 for ARGoS by 
 1.6.1
